# Quality and availability of information in primary healthcare: the patient perspective

**DOI:** 10.1080/02813432.2020.1718311

**Published:** 2020-01-31

**Authors:** Tobias Abelsson, Helena Morténius, Stefan Bergman, Ann-Kristin Karlsson

**Affiliations:** a Department of Research, Education and Development, Region Halland, Halmstad, Sweden;; b Primary Health Care Unit, Department of Public Health and Community Medicine, Institute of Medicine, The Sahlgrenska Academy, University of Gothenburg, Gothenburg, Sweden;; c Department of Health Care, Region Halland, Halmstad, Sweden;; d FoU Spenshult, Halmstad, Sweden

**Keywords:** Emotions, health literacy, information dissemination, information literacy, information seeking behavior, primary health care, qualitative content analysis

## Abstract

**Objective:** To explore lived experiences of patients communicating with and receiving information from primary health care.

**Design:** Qualitative study analysing transcribed interviews by descriptive content analysis.

**Setting:** Recruitment and interviews took place in southern Sweden in three primary care centres where privacy and undisturbed interview environments was ensured.

**Subjects:** 17 primary care patient informants, 9 men and 8 women aged 31 – 84 years with varying educational levels from primary school to post graduates.

**Main outcome measures:** Thematic categories and subcategories reporting the lived experience of the patients.

**Results:** The analysis yielded three categories and identified as a main theme a feeling of unpredictability based on the emotional aspects of feeling lost and vulnerable when trying to access primary care. The category” Need for easy access” illustrated emotional aspects of importance to patients when contacting primary health care.” Need for individual adaptation” described the need to individually adapt health related information.” Information exchange” comprised experiences of information evaluation and understanding new information.

**Conclusions:** Patients generally trusted the information received, but experienced a lack of communication, which evoked feelings of unpredictability and abandonment. Experiences of limited access to primary health care and the need for varying degrees of adaptation on the part of the individual were factors of concern for how patients experienced the care.

**Key Points**
Smooth communication and understandable information are fundamental for quality primary health care. This qualitative interview study identified the following key points from analysing the views of 17 patients:
• Patients indicated a feeling of unpredictability due to lack of access to and communication with health professionals.
• Patients sometimes reported an inability to understand information conveyed by health professionals.
• Being able to form relationships with health professionals was crucial for patients’ trust and understanding.

## Introduction

At some stage of life most people will become a patient, making the relationship with health professionals (HPs) a reality. When the need occurs, modern society promotes the patient’s right and ability to influence her/his own care [1]. This expectation can constitute a burden for the individual, who has to make complex and sometimes life changing decisions based on new information, the source and quality of which are unknown [2]. The abundance of information on health and illness available today makes it easy for everyone to access such information [1,2].

As information and communication are fundamental concepts in this article, a short definition might be helpful. We primarily define information as any knowledge pertaining to an individual’s health and/or care conveyed by any means by primary health care (PHC) or other sources. Communication is a process of mutual influence among people, where information serves as the content [3,4].

To explain the process of informing oneself we turn to Kuhlthau’s information seeking process (ISP) [5], which describes user experiences, feelings and actions in their effort to meet their need for information. According to the ISP, an individual searching for information starts with vaguely formulated notions of what to look for. As more information is gradually revealed, her/his level of knowledge increases, thus enabling more focused information requests [2,5,6]. In a patient-PHC setting the information need resides within the patient due to her/his lack of knowledge about a certain condition or other health-related issues [5]. In order to meet this need the patient turns to PHC, thus beginning an information seeking process targeting HPs within PHC, as they are often the preferred information source.

In order to integrate and act upon the information found, which is the final phase of ISP [5], the patient requires a basic knowledge of how to find, evaluate and make use of information. This skill set is defined as information literacy [7–9].

A more specialized term, health literacy, is used for the health care aspects of information seeking. Although similar to information literacy, health literacy includes a prospective aspect; the ability to use information to gain knowledge of how to improve one’s health, evaluate information and apply it in order to avoid illness [10–12].

The meeting between self-informed patients and HPs places demands on the individual HP, such as being communicative [1] and up to date on the latest findings and advances in their field of expertise [2,12–16]. The HP is the information provider, while the patient is the recipient and sometimes has to assimilate life changing information. When given a choice, most patients prefer human contact to online health services and generally have a strong desire to make health-related decisions in cooperation with a HP [14,16,17].

When these options are not available, the individual response might be anxiety or frustration. Studies suggest that patients tend to turn to alternative sources when experiencing a lack of confidence in or dissatisfaction with the information given by the HP [2]. This in turn can trigger a spiral of information searches, second opinions and in some cases even obtaining a desired treatment privately [1,15,18].

Evidence indicates that good quality information provided in a way that the patient can understand has a number of positive effects for her/him [12,16,19]. Essential for such communication is a good personal relationship between patient and HP characterized by an understanding of the patient’s social context, expectations and experiences. [12,13,16]. Receiving information in such a positive way not only helps the patient to adjust her/his expectations by evoking a sense of security but can also counteract unjustified expectations caused by incorrect information obtained from other sources [16,17,19,20].

Concerning patients’ emotional experiences, studies are available that illustrate the need for continuity of information and HPs for patients who are involved in a complex chain of healthcare services [18]. Continuity means that patients experience security when treated by the same HP and not confronted with “new faces” [18]. Likewise, some studies demonstrate the importance of individual adaptation and care for patients in stressful situations such as impending major surgery [19]. Treating the patient as an individual and providing information in a manner that facilitates easy assimilation also seems to help her/him to adjust outcome expectations [16,18,19].

There is also research focusing on the general population, health consumers’ need for information and information habits [17,21,22]. A Japanese study about health consumers’ information habits found that the use of information differed somewhat in relation to factors such as age, educational level and sometimes geographic location [17]. The main reason for searching for information was to obtain confirmation or moral support in a health-related matter [14]. When it came to actual medical decisions, patients preferred to talk to another human being, either a HP or a peer [1,17,23].

The Cochrane foundation compiled research findings on improving patient communication in a systematic review, which concluded that evidence-based interventions aimed at educating patients to better communicate with HPs had little or only limited success [20].

There is a need for complementary research on communication related to the emotional needs of patients in a PHC setting in order to improve the quality of information and patient communication. Previous research is somewhat dated and does not take account of information dissemination in a PHC setting. Thus the aim of this study was to explore patients’ lived experiences of communicating with and receiving information from PHC.

## Method

### Study design

The study has a descriptive, qualitative design. It is based on individual interviews carried out by means of a content analysis method as described by Graneheim and Lundman [24].

### Sample

Recruitment of strategically selected informants took place in three different public PHC centres in southern Sweden from August 2017 to April 2018. The intention was to collect data from about 20 informants to achieve saturation. The recruitment was planned to be performed by the help of medical assistants employing their knowledge of the patients. However, this was found to be practically impossible because the allocated resources in the centres varied during the study. It was therefore deemed necessary to change the recruitment method at about the halfway stage to the use of volunteer forms. The result of this was split recruitment with 11 informants volunteered via recruitment forms and 6 with the help of the medical assistants. As these complications during recruitment threatened to weaken the methodological integrity of the study and reduced the prospect of completing it within a reasonable time, a decision was made to stop at 17 informants. The amount of data collected was still deemed sufficient for a content analysis.

The inclusion criteria were informants aged at least 18 years and able to speak and understand Swedish. The final study population comprised 17 patients, 9 men and 8 women of Swedish origin, aged between 31 and 84 years, mean age 65 years. 11 of the informants were recruited from PHC centres located in an urban setting and 6 from a PHC centre located in a rural area. Informants with the lowest educational level had graduated from Swedish primary school, but often complemented their education later in life. The most highly educated informants had obtained doctoral degrees.

### Setting

There were two settings in which the participating PHC centres were geographically located. An urban area denotes a city with a population of more than 20,000 local access to PHC and more specialized medical service. A rural area indicates towns with only a couple of thousand inhabitants but with local access to PHC.

### Data collection

The patients were first informed about the study when they visited one of the three participating PHC centres. At one location the receptionists informed them about the study and asked if they were willing to participate. At the other two, patients volunteered by filling in a participation form that contained contact information, age and educational level. The completed forms were placed in a sealed box, which was later collected by the first author (TA). All volunteers that fitted the study then received an introductory letter with more detailed information about the study. A consent form and an envelope for returning it to TA were also enclosed. When the informants had returned the consent form, they were contacted by telephone in order to arrange the time and place for the interviews, which were conducted in the PHC centres or the first author’s office. Care was taken to ensure privacy and a disturbance free environment for the interview.

The interviews were performed by TA with the aid of a written interview guide (Appendix 1). The interview started by asking the patients to describe their general thoughts about and experiences of communicating with and obtaining information from PHC. The patients were then requested to share their thoughts on their evaluation of the information received from PHC, both face-to-face and via other modes of communication. In addition, the retrieval of information about medical issues and the patient’s experiences of trust in the sources of information were also covered. Each question was followed up by attendant questions in order to clarify and deepen the interview. The average duration of the interviews was 30–45 min. The interviews were recorded and transcribed verbatim by TA.

### The research team and preunderstanding

The research team consisted of the following occupational categories: Hospital librarian, Medical and healthcare social worker, Healthcare strategist and General practitioner. All members have long experience of encountering patients. The combined expertise of the research team ensures a preunderstanding of building relationships with patients as well as of the clinical context in which the encounter takes place. There was no pre-existing relationship between any of the patients and the members of the research team.

### Data analysis

Data analysis in accordance with the method presented by Graneheim and Lundman [24] consisted of six steps: 1) The complete transcripts of the interviews were read by the first author in order to become acquainted with the material. The co-authors AKK and HM each read and identified the meaning units in five transcripts. 2) Meaning units relevant to the aim of the study were identified by the authors and discussed until consensus was achieved. 3) Meaning units were condensed and coded. 4) The coded material was then grouped into subcategories and categories. The authors discussed the categories until agreement was reached, which constituted the manifest meaning of the study. 5) The latent meaning of the study was formulated into a main theme in cooperation between the authors. 6) Finally, the result was written down and illustrated by quotations from the interviews. The goal of the parallel analyses conducted by the co-authors was to enhance reliability and ensure quality. The following tables illustrate the analysis process by presenting an overview of the categories and theme (Table 1) and the coding process (Table 2).

**Table 1.** Overview of the result structure.

Subcategory:	Category:	Theme:
Contact paths	The need for easy access	The feeling of unpredictability
Administrative challenges		
Patient involvement	The need for adaptation	
Barriers to understanding		
Continuity		
Information evaluation	Information exchange	
Information quality		

**Table 2.** Examples of the analysis process.

Meaning unit	Condensed meaning	Code	Subcategory	Category
They need to take more long term responsibility and follow up each case. If it have been any progression.	Missing long term commitment by the HP.	Long term commitment	Patient involvement	The need for adaptation
When you arrive here you are under the impression that you are in good hands and receive care that is up to date. But it is not quite like that.	Noticeable difference in knowledge amongst HP.	Noted knowledge difference	Information evaluation	Information exchange

### Ethical approval

The study was approved in 2017 by the Research Ethics Committee of the University of Lund, Lund, Sweden (No. 2017/281).

### Methodological considerations

To the best of our knowledge this is the first study to explore the emotional aspects of obtaining information from PHC. Using qualitative methodology, the study investigated the information dissemination process with focus on the emotional response of patients. This focus contributed knowledge of the complexity that makes patient information and communication such a multifaceted topic.

The change of recruitment method midway into the study is not optimal although necessary. Besides this change, no further changes were made and the context remained unchanged. All volunteers were notified about the study when they visited the PHC centres due to a pre-existing or new clinical issue. All patients who met the inclusion criteria had an equal opportunity to volunteer either by filling out the form or being invited to participate by the reception staff.

The change in the recruitment strategy appears to be reflected in the number of patients. It seemed that patients found it easier to agree to participate in the study if asked by another person rather than having to fill out a form. However, the influence of this factor is unclear, as the personal connection between the medical assistants and the patient could constitute either an encouragement or a deterrent. Filling out a form could be beneficial for those patients who did not wish to be directly confronted by the question of participation. Furthermore, this recruitment method was dependent on potential informants reading a notice in the waiting area of the PHC centre, which might have reduced the number of informants. Nevertheless, both methods served as a first contact between patients and the first author and as such fulfilled their purpose.

The characteristics of the patients could have been somewhat more influenced when using the first method, as the medical assistants would have had the opportunity to actively pursue patients they considered suitable. This potential bias was eliminated when using the forms. As the final number of informants included all patients who volunteered, it would probably have been necessary to change to a more active recruitment strategy in order to achieve a larger sample.

There are several methods for analysis of transcribed data. The main reason for choosing content analysis was that it is recognized as one of the best methods for analysing transcribed material such as the interviews on which this study is based.

The strength of this study is its focus on information and emotion in connection with the care process. Such a holistic perspective might help to deepen understanding, thus paving the way for better patient – HP communication in PHC.

Readers should bear in mind that the study results are based on data collected from a small number of informants. The study is set in PHC and the results may vary in another setting, such as prehospital or hospital settings.

## Results

The latent meaning that emerged from the analysis was based on a main theme constructed from the manifest meaning of the three main categories and seven subcategories, each of which describes nuances of the main theme.

### The feeling of unpredictability

The data revealed a main theme labelled The feeling of unpredictability. This was evident throughout the interviews, as patients reported being unable to rely on meaningful interaction with the HPs at their PHC, resulting in a lack of understanding and a feeling of unpredictability. These impressions varied from a mild sense that certain details in one’s case were disregarded to a more immediate need for self-management in one’s own care to get things moving.

### The need for easy access

Encompassing the informants’ experiences of the difficulties involved in contacting PHC. This category could be broken down into the subcategories Contact paths and Administrative challenges:

### Contact paths

The preferred way of contacting the PHC centre was the internet followed by the telephone. Informants expressed satisfaction with the call management system and the possibility to book appointments *via* the telephone. In addition, the Swedish online health counselling service 1177 also emerged as a frequently used method of contact. Regarding functionality, the informants experienced that the emergency care functioned well, although there was criticism of the difficulty in making appointments with their doctor and contacting the “right” persons in order to obtain the desired results.

As I said before, if you can communicate properly with the nurses, all is well and you can get the help you need when you visit but getting help later is more difficult, these are things that need to be improved.

Patient F, male, 81 years

### Administrative challenges

Some informants had experienced rigidity or inertia when requesting feedback from PHC. Informants also mentioned general tardiness in communicating results and follow-ups of a previous visit, in addition to the difficulty of correcting clerical errors.

…but it was hell before they recognized that I was right and it was changed. But it is still not right because I will return this week for a new test…

Patient B, male, 77 years

### The need for adaptation

The category comprises the informants’ experiences of how they are taken care of as individuals and how the care is adapted to their own needs. The subcategories Patient involvement, Barriers to understanding and Continuity were identified.

### Patient involvement

According to several informants, a positive and very important factor was the feeling of being noticed and cared for as an individual. This involved the way they were greeted and informed upon arrival at the PHC centre and encompassed the whole visit. While discussing this subject, patients mentioned that a positive factor was when the HP they encountered was ´present´. Although being present was generally positive, there appeared to be a difference in how much information each HP provided. A few expressed a feeling of having to be ´on your toes´ and actively engage in their own care or seek out information that might ´get stuck´ somewhere in the healthcare system.

… You yourself have to plan ahead when it comes to where you seek medical care, because I wrote a self-referral to the orthopaedic department, but I should in fact have sent it to Carlanderska or the spinal centre in Gothenburg.

Patient C, female, 72 years

### Barriers to understanding

Informants expressed that many of the HPs they encountered lacked Swedish language skills. However, some also mentioned being unable to understand the HP even when she/he spoke fluent Swedish. Another issue that led to confusion was HPs who questioned the need for the medicines prescribed by one of their colleagues but did not bother to conduct a proper investigation.

On the last two visits, relatively recently, there were some linguistic problems.

Patient G, female, 42 years

### Continuity

Almost all informants voiced their concerns about meeting new staff members more or less every time they visited the PHC centre. Being recognized by the HP and vice versa was described as essential for feeling secure and cared for. Greater attention to the patient’s situation and her/his previous medical history was also described as important but lacking.

There is a new doctor every time. My feeling is that, well… If they prescribe some pills and take a chance, then it’s no longer their problem.

Patient E, male, 68 years

### Information exchange

This category encompasses the informants’ experiences related to obtaining information and deciding on its value and relevance. It consist of the subcategories Information evaluation and Information quality.

### Information evaluation

In addition to the PHC centre there seemed to be three main sources for obtaining trustworthy information. Some informants stated that their main source of health information was the internet and Google. Others preferred to phone the medical information service 1177, while the third source of information was family and friends. There were also statements about the need to double check information received from HPs.

I have googled to check that the information my doctor gave me is the same, then I feel that I can trust it automatically.

Patient G, female, 42 years

### Information quality

Collected here are the individual factors which, in the opinion of the informants, ensure good quality information and trustworthiness. Generally informants encountered HPs who acted in a manner that made them appear trustworthy and reliable. However, a few also noted that the degree of knowledge sometimes differed between individual staff members.

You notice it. I suppose it depends on how much experience they have. You notice at once those who are just waffling.

Patient D, male, 72 years

## Discussion

In this qualitative interview study of primary care patients in Sweden we found that communication and information issues resulted in what could be summarised as a sense of unpredictability among the informants as presented in the main theme of our content analysis.

### Main findings

The main theme was derived from seven subcategories structured into three main categories and labelled: "The feeling of unpredictability". The informants were generally willing to rely on the information given by HPs but this trust was weakened by the difficulty accessing it. Basic PHC was generally experienced as functional but slow. Having a good personal relationship with an individual HP was reported to positively influence the chance of making progress. On the subject of adaptation, positive feelings were experienced when patients felt recognized as an individual and the HP focused on their specific case. Health information was obtained from three main sources; Internet/Google, the Swedish online health counselling service 1177 and family/friends. Some felt the need to double check the information received from the HP. The most frequent emotions concerned the experience of being exposed, frustrated and sometimes feeling abandoned by the healthcare system.

### General discussion and results

Our results describe the informants’ desire to be able to trust their HPs and the healthcare system. However, almost all informants reported a lack of continuity and communication in addition to feeling confused when trying to access PHC. Although the aim of the interviews was to explore patient experiences of communication in the PHC context, the first issue almost always mentioned was the difficulty accessing the PHC centre. They also expressed that satisfaction was negatively influenced by what they perceived as an inherent systemic inertia in PHC that impacts on aspects such as follow-up and receipt of test results.

The informants in this study underlined the need to be seen as an individual and experience continuity in the context of their own situation. This fact is supported by previous research, which states that a HP who clearly explains the individual’s health situation is more likely to facilitate the patient’s understanding [13,16].

HPs’ communication skills are under scrutiny every time they meet a new patient with whom they are supposed to build a relationship of trust [1,13]. The present study identified difficulties encountered by patients when trying to enter into such a relationship. The patients stated that language skills were lacking, or in a worst-case scenario, close to non-existent. The informants also mentioned that the HP could create a feeling of stress by reducing the time spent on each consultation to a minimum in order to maximize efficiency and time utilization. This often resulted in a feeling of being misunderstood by, or developing an aversion towards, the HP. Lack of trust can lead to non-adherence to prescribed therapies [13,25] because the patients either do not understand what to do, or feel that the information received is not trustworthy. The importance of a good relationship with the patient that enables HPs to build trust and adapt to the patient’s information processing ability in complex situations has been highlighted in earlier studies [2,13,16,18,19,21,25].

According to Kuhlthau’s ISP-model, feelings of confusion and anxiety are common in the early stages of an information seeking process [5,6]. It is evident that patients have an emotional need when seeking medical attention, which is just as important as the physical need. As the results of this study show, HPs generally tend to focus more on patients’ physical treatment rather than the emotional aspect of why they seek help. If patients are unable to satisfy both needs they will strive to remedy the situation by seeking out whatever information they can from alternative sources [1,2,15].

When searching for alternative information, a person always runs the risk of finding controversial or even misleading information. Therefore, one should be alert to the fact that a great deal of information may not be trustworthy [1,12,15,16]. Knowledge is required to identify good research and trustworthy sources, which is a fundamental skill necessary for both HPs and patients seeking health information [7,8]. Irrespective of their educational level, the informants in our study seemed to rely strongly on information from the internet to confirm information received from HPs or to search for a possible diagnosis themselves.

Among informants who stated that they used the internet as a main source, many mentioned first turning to the Swedish online health counselling service 1177. Some informants stated that they used the internet to search for symptoms, thus obtaining information on possible conditions, about which they subsequently asked their HP. This strengthens the image of patients as curious information seekers employing multiple sources. It also underlines their wish to trust official guidelines when in doubt and before deciding on a course of action [1,26].

There are indications that the level of health literacy is correlated with other factors such as educational level and degree of information literacy [17,27]. In the present study there might also be a difference linked to factors such as social network and educational level in the degree to which the informants adapt information. Some of the informants stated that before seeking medical care in non-emergency cases, they mostly placed their trust in the opinions of family and friends. This need for security in the form of conferring with others can be seen in previous studies in many different areas of communication research [17,22,23].

## Conclusion

Overall, the informants expressed a high level of trust in PHC when discussing it in general terms but had concerns about the quality and attention to detail in their own case. The main concern of almost all informants seemed to be a missing prerequisite for effective communication: easy access to the PHC centre and HPs. Lack of communication appeared to evoke a feeling of needing to fend for oneself in order to obtain information and relevant care. The lack of both continuity and information transfer between patients and a number of different HPs was identified as a cause of uncertainty and vulnerability.

Although information provided by the HPs was generally believed to be trustworthy, some patients also turned to the internet in order to verify or obtain alternative information. Patients generally stated that friends and family members were reliable resources for health information in addition to HPs and 1177. This mix of emotions; confusion, feelings of vulnerability, abandonment and not receiving adequate information, resulted in a feeling of unpredictability that triggered doubt about PHC and its ability to provide trustworthy care.

## Clinical implication

A complete resolution of the complex problems associated with communication that leads to patient satisfaction is beyond the scope of this study. However, we have identified the need for continuity in PHC, which could serve as the foundation for developing communication and information methods that cater to the individual. If such methods were introduced, it would probably enhance patient safety, financial efficiency and free up PHC resources. Concrete recommendations for starting this journey would be for PHC to be more active in offering alternative sources of information in order to promote patient involvement and eliminate some sources of anxiety.

## Further research

Further research could involve the investigation of HPs’ experiences of informing patients and keeping up to date as professionals. This might open up possibilities to investigate processes that ensure the use of high-quality information in patient care.

## Appendix 1

Interview guide

1. What are your general thoughts about information and communication in primary healthcare?
2. Keeping the concept of information in mind, what would you say, signifies proper reception and good care?
3. Can you describe anytime when there has been any kind of difficulties in your contacts with primary healthcare?
4. How do you experience the distribution of responsibilities between patient and primary care?
5. How do you proceed if you need to know something related to health or healthcare?
6. There are three concepts connected to care that I would like to discuss:
  1. How would you describe the Credibility in primary healthcare?
  2. In which way is the scientific involvement visible in primary healthcare?
  3. Can you describe your experience of the HP:s process of searching for, evaluate and finally implement new information, in your own care process?
7. Do you have any questions regarding this interview?

Demographic questions:

- What's your occupation?
- What is your educational background?
